# Tissue Engineering in Liver Regenerative Medicine: Insights into Novel Translational Technologies

**DOI:** 10.3390/cells9020304

**Published:** 2020-01-27

**Authors:** Zahra Heydari, Mustapha Najimi, Hamed Mirzaei, Anastasia Shpichka, Marc Ruoss, Zahra Farzaneh, Leila Montazeri, Abbas Piryaei, Peter Timashev, Roberto Gramignoli, Andreas Nussler, Hossein Baharvand, Massoud Vosough

**Affiliations:** 1 Department of Stem Cells and Developmental Biology, Cell Science Research Center, Royan Institute for Stem Cell Biology and Technology, ACECR, Tehran 1665659911, Iran; zahrabiology85@gmail.com (Z.H.); zahrafarzaneh2006@yahoo.com (Z.F.); 2 Department of Developmental Biology, University of Science and Culture, ACECR, Tehran 1665659911, Iran; 3 Laboratory of Pediatric Hepatology and Cell Therapy, Institute of Experimental & Clinical Research, Université Catholique de Louvain, B-1200 Brussels, Belgium; mustapha.najimi@uclouvain.be; 4 Research Center for Biochemistry and Nutrition in Metabolic Diseases, Kashan University of Medical Sciences, Kashan 121135879, Iran; h.mirzaei2002@gmail.com; 5 Institute for Regenerative Medicine, Sechenov University, 119146 Moscow, Russia; ana-shpichka@yandex.ru (A.S.); timashev.peter@gmail.com (P.T.); 6 Siegfried Weller Institute for Trauma Research, University of Tübingen, 72076 Tübingen, Germany; marc.ruoss@student.uni-tuebingen.de (M.R.); andreas.nuessler@med.uni-tuebinge (A.N.); 7 Department of Cell Engineering, Cell Science Research Center, Royan Institute for Stem Cell Biology and Technology, ACECR, Tehran 1665659911, Iran; montazeri.leila@gmail.com; 8 Department of Tissue Engineering and Applied Cell Sciences, School of Advanced Technologies in Medicine, Shahid Beheshti University of Medical Sciences, Tehran 1985717443, Iran; 9 Department of Biology and Anatomical Sciences, School of Medicine, Shahid Beheshti University of Medical Sciences, Tehran 1985717443, Iran; 10 Department of Polymers and Composites, N.N.Semenov Institute of Chemical Physics, 117977 Moscow, Russia; 11 Department of Laboratory Medicine, Division of Pathology, Karolinska Institutet, 171 77 Stockholm, Sweden; roberto.gramignoli@ki.se; 12 Department of Regenerative Medicine, Cell Science Research Centre, Royan Institute for Stem Cell Biology and Technology, ACECR, Tehran 1665659911, Iran

**Keywords:** tissue engineering, regenerative medicine, liver, translational medicine

## Abstract

Organ and tissue shortage are known as a crucially important public health problem as unfortunately a small percentage of patients receive transplants. In the context of emerging regenerative medicine, researchers are trying to regenerate and replace different organs and tissues such as the liver, heart, skin, and kidney. Liver tissue engineering (TE) enables us to reproduce and restore liver functions, fully or partially, which could be used in the treatment of acute or chronic liver disorders and/or generate an appropriate functional organ which can be transplanted or employed as an extracorporeal device. In this regard, a variety of techniques (e.g., fabrication technologies, cell-based technologies, microfluidic systems and, extracorporeal liver devices) could be applied in tissue engineering in liver regenerative medicine. Common TE techniques are based on allocating stem cell-derived hepatocyte-like cells or primary hepatocytes within a three-dimensional structure which leads to the improvement of their survival rate and functional phenotype. Taken together, new findings indicated that developing liver tissue engineering-based techniques could pave the way for better treatment of liver-related disorders. Herein, we summarized novel technologies used in liver regenerative medicine and their future applications in clinical settings.

## 1. Introduction

The liver plays a crucial role in different physiological functions such as protein, carbohydrate, and lipid metabolism, detoxification of xenobiotics, storage of glycogen and vital biomolecules, production and excretion of bile and cholesterol compounds, synthesis of albumin and clotting factors, ammonia detoxification and more [1]. In this regard, failure of liver functions may lead to a wide range of liver dysfunction conditions with different levels of severity, morbidity or mortality [2]. According to world health organization (WHO) reports, liver diseases are the 12th cause of mortality globally and their incidence and prevalence are increasing. Among liver disorders, nonalcoholic steatohepatitis (NASH) and cirrhosis are common [3].

Hepatocytes are liver parenchymal cells, while cholangiocytes, endothelial cells, tissue-resident macrophages (Kupffer cells), and stellate cells are known as the liver non-parenchymal cells. An active, efficient interaction between hepatocytes and the surrounding cells and/or microenvironment is necessary for their maintenance and proper function [4]. A healthy liver shows excellent regenerative capacity in response to injury. Such ability can be significantly impaired when the liver faces a severe acute injury or an extreme chronic disorder which is commonly associated with progressive liver inflammation and fibrosis [5]. Orthotopic liver transplantation (OLT) is the “gold standard” and the only established treatment for end-stage liver diseases and acute liver failure. The OLT faces several limitations and obstacles including the shortage of donated organs, post-operational complications and high hospitalization costs as well as the need for a life-long immunosuppressive therapy [6,7]. Therefore, due to the aforementioned limitations, alternative therapeutic approaches are necessary to bridge or replace affected organ. Cell-based therapy is a novel therapeutic approach that was introduced several years ago for treatment of different hepatic pathologies [8,9,10,11], and inherited metabolic and congenital liver disorders [12,13].

Infusion of mature isolated primary hepatocytes is not the only promising strategy for liver regeneration. Liver regenerative medicine is an interdisciplinary field that aims to develop novel platforms for disease modeling and therapeutic approaches for liver diseases in order to regenerate, repair or replace damaged organs [14]. Recently, by using microfabrication and nanotechnology, novel in vitro modeling of liver micro-tissues was described; it resulted in better understanding of cellular and molecular mechanisms of liver regeneration [15].

TE and regenerative medicine are growing side-by-side in medical discipline, deeply interconnected by progressing research on stem cell biology, gene editing technology, synthesis of bio-functional scaffolds, nanotechnology and intelligent 3-dimensional (3D) bio-printing devices [16]. Typically, in TE, combination of live cells and defined matrices with bioactive factors usually form implantable constructs with physiological functions [16].

In this review, we summarized and highlighted various applications of TE in liver regeneration. Then we focused on different scaffolds which are used in TE and explained different approaches in liver TE. Finally, extracorporeal liver devices and in vitro and in vivo models used in liver TE were discussed.

## 2. Application of Tissue Engineering in Liver Disease

To restore liver functions using a functional implantable liver tissue, the following three components are essential: i) bio-compatible scaffolds, ii) functional cells which could be derived from adult tissues or from pluripotent stem cells [17,18] and iii) standardized growth factors and active bio-molecules.

**Figure 1:**
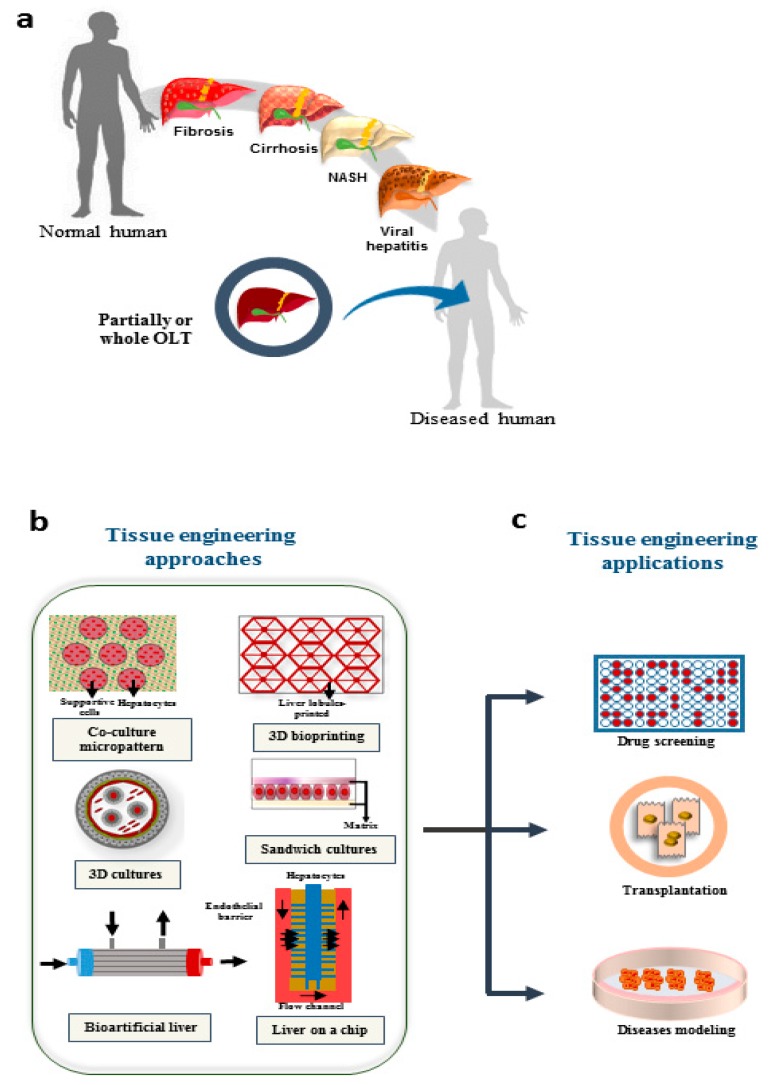
Possible applications of TE in treatment of liver diseases. (**a**) Different diseases that result in liver failure; the only approved approach for end stage diseases is liver transplantation. (**b**) Different engineering approaches are growing to overcome the limitations in treatment of organ failure, drug screening, and disease modeling. (**c**) The possible applications which are promising using tissue engineering approaches. NASH: Nonalcoholic steatohepatitis; OLT: orthotopic liver transplantation.

The progress in implantable engineered hepatic tissues could be a promising strategy to overcome the current limitations of cell-based approaches. Limited cellular engraftment and short-term survival of implanted cells are the major challenges which are yet to be achieved [19]. Diverse methodologies can be employed to produce hepatic micro-tissues, such as cell encapsulation, 3D printing, microfluidic systems and decellularization/recellularization approaches [20]. A schematic representation of liver diseases, and possible application of technologies used in liver tissue engineering presented in Figure 1.

The progress in implantable engineered hepatic tissues could be a promising strategy to overcome the current limitations of cell-based approaches.

## 3. Different Scaffolds Applied in Liver TE

Biocompatibility was the significant feature of the first-generation biomaterials; however, second-generation ones are characterized by their bio-interactivity. While the first-generation biomaterials were passive, second generation components were specifically designed to induce tissue regeneration. In order to improve the mechanical features of polymers, to use their great properties and to enhance tissue interaction, ceramics and polymer composites have been proposed [21]. Nowadays, third-generation biomaterials are bio-responsive and capable to activate specific genes involved in cell differentiation, function and proliferation [22].

### 3.1. Physical and Biochemical Properties of Scaffolds Used in Liver TE

Safety and biocompatibility are principal features of crucial importance for biosynthetic liver scaffolds. Cells embedded in bio-artificial scaffolds should be capable to replicate, dig for extra space or even generate new extra cellular matrix (ECM) [23,24]. Thus, an ideal scaffold should mimic the physiologic properties of native liver ECM. However, the development of a scaffold, capable to support cell functions depends on parameters such as the surface features, underlying material, and characteristics of the selected cell line [23]. Biocompatibility of scaffold permits concurrent generation of new tissues along with matrix degradation [25]. The biological characteristics of the scaffolds influence their interactions with target organs and tissues. Furthermore, an optimized scaffold should circumvent immune system for incorporated cells. The immune-inert biomaterials with characteristic immune regulatory features (i.e., reduced activity of NK cells as well as B and T cells-mediated immunity) have been recently proposed [25].

The majority of scaffolds are typically made of hybrid materials, bio-ceramics and polymers, whether synthetic or natural [26].

In order to achieve better cell attachment and make the surface more similar to the in vivo conditions, 3D culture systems containing ECM proteins have been proposed.

While hydrogels such as the collagen sandwich or Matrigel consist of almost exclusive ECM proteins, they are often integrated directly into the scaffold matrix in scaffold-based 3D cultivation systems, or the scaffold is subsequently coated with them. In addition to the liver-specific ECM proteins, fibronectin, collagen type I and gelatin are often used [27,28]. Since gelatin is a byproduct of collagen hydrolysis, it contains the same RGD peptides. Therefore, it is also often used as a component of the scaffold matrix [29].

Pre-incubation of the scaffold in serum-containing medium, may enhance cell adherence. In order to achieve optimal cell adherence, a relatively long pre-incubation period of up to 10 days, is sometimes required [30].

In general, scaffold-based 3D cultivation systems which are commonly used for the cultivation of liver cells, can be divided into two groups. The first group includes porous scaffold materials such as Cryogels^®^, porous natural products, or scaffolds made using electrospinning or a 3D printer [31,32,33]. In the second group, the live cells are completely enclosed by the scaffold matrix [32]. Among the 3D culture methods, many systems use hepatocytes on natural sponges or other natural products like silk fibroin protein [1,30,32,34].

### 3.2. Elasticity, Porosity, and Other Physical Properties of Scaffolds in Liver TE

The optimum stiffness and elasticity of a healthy human liver have been estimated between 400 and 600 Pa [35,36]. The liver lobules have no basal membrane and relatively little amount of extracellular matrix. Together with the numerous fenestrations and gaps within sinusoidal endothelial cells, this structure allows rapid bidirectional exchange of macromolecules between plasma and hepatocytes [37]. For a bio-artificial scaffold, at least a porosity of 95% is required to allow the exchange of nutrients and wastes products. Additionally, a large surface/volume ratio is necessary to promote hepatocyte attachment and maintenance [28]. The optimal pore size is required to maintain the polarity of the cells. It seems that cell-cell interactions are also required, which suggests rather larger pores. In a study carried out on rat hepatocytes, it has been shown that the pore sizes of 10 µm or 80 µm lead to an improved hepatic function. Moreover, an increase in metabolic function with the 80 µm pores was observed especially at a high cell concentration, which indicates that an interaction among the cells [38]. In order to provide a sufficient supply of nutrients and to allow facilitated exchange among the cells, the scaffold material should be highly permeable. Since there is no blood supply in vitro, it is necessary to reduce the number of cells particularly in static culture condition compared to the in vivo conditions. However, it should be considered that a reduction in the cell concentration also reduces the possibility of cell-cell interactions, which is accompanied by a reduced function of the cultivation system. In order to ensure that the cells can efficiently migrate through the scaffold, it is also necessary that the pores should be interconnected. The stiffness of the scaffold also has an influence on the metabolic activity of the cells. In a recent study, polydimethylsiloxane (PDMS) was used to generate different levels of rigidity, resulting in cells with superior metabolic activity when cultured on a substrate with approximate 2 kPa stiffness compared to 50 kPa on polystyrene substrate, where the stiffness can be approximately 3 GPa [39].

Synthetic hydrogels can be degradable or non-degradable. Compared to natural ones, the advantages of synthetic hydrogels increased the potential application of synthetic hydrogels in TE approaches. These hydrogels are reproducible, less immunogenic, mechanically tougher. But these hydrogels are not popular for liver tissue engineering in clinical application [40].

Table 1 shows some common 3D models which employ scaffold or hydrogel type and their advantages and disadvantages considering the culture method.

## 4. Different Approaches in Liver TE

### 4.1. Decellularization/Recellularization Approach

Extracellular matrix plays a key role in cell adherence, polarity, proliferation, differentiation [47,48] and can promote liver functions such as cytochromes P450 (CYPs) activity in organoids [49].

Due to polymorphic differences that exist between human and other species, the ideal biomaterials for liver tissue engineering should be human derived. Decellularized organ is a suitable scaffold with a proper and specific microstructure for the implanted cells of the original organ.

By using the ECM of an acellularized liver, which can be integrated into the scaffold matrix, the highest similarity with the in vivo conditions can be achieved [50]. During decellularization, cells and other immunogenic factors are removed and only the natural scaffold of tissue remains. This approach could provide an alternative source of implantable organs in OLT [51]. Compared to other techniques, in this method, the original template of the vascular network and biliary system are maintained, and this is the greatest point that can be noted in liver tissue engineering [51]. In fact, three important parameters should be considered in recellularization technique including; i) selection of suitable cell types, ii) route of cell administration, and iii) optimized cell seeding protocols. [20]. In 2015, the first whole organ decellularization protocol was introduced by Ott et al. [52].

This decellularized human livers were later repopulated using hepatic stellate cells (LX2), hepatocellular carcinoma (Sk-Hep-1) and hepatoblastoma cells (HepG2). Ex vivo preservation was prolonged for up to 21 days, with excellent cell viability, motility and proliferation and remodeling of the extracellular matrix [44]. Another study developed a humanized liver by using acellularized porcine liver and combinations of human fetal hepatocytes and stellate cells. This study demonstrated that the acellularized matrix could support and induce phenotypic maturation of engrafted human fetal hepatocytes in a continuously perfused system [53].

An efficient and successful decellularization process, preserves the initial pattern of ECM and provides a proper niche for seeded cells. After cell homing, the neo-organ should be able to present some levels of functional maturation in the perfusion bioreactor and subsequently, the new organ could be transplanted without extreme immunosuppression. In 2010, Uygun et al. published the first study on recellularization of an acellularized liver that was transplanted in a rat model. The recellularized graft was maintained in a perfusion chamber for up to 2 weeks before implantation. This was the first report that supported functionality of the re-seeded hepatocytes which were cultured on a decellularized 3D ECM scaffold. However, this study highlighted some key questions; for instance, what is the proper flow rate for recellularization?

At slow flow rate, the reagents do not reach the depth of tissues and at fast flow rates, cell clamps are produced. Furthermore, during recellularization, intravascular liver thrombosis could be happening, and the vessels might be blocked [54].

To examine the best method for recellularization of an organ, a study from 2011 described as a multistep infusion is associated with the most favorable results. The described methods are as the following: i) direct parenchymal injection, ii) continuous perfusion and iii) multistep infusion. This study showed that multistep infusion is associated with the most favorable results [55].

In conclusion, different bio-scaffolds can be employed in transplantation and pharmaceutical and toxicological studies and may act as a reliable tool to study normal organ development as well as liver basic pathology [56].

### 4.2. Cell Encapsulation Techniques in Liver TE

Encapsulation is an advanced technology for immobilization of allogenic or xenogeneic cells in a semipermeable scaffold in order to escape immune system and deliver biological products to patients without any immunosuppression [57]. While many advanced technologies are under development, cell encapsulation is the only approach that currently meets all the essential prerequisites for a truly translational medicine [58]. An acceptable capsule should be biocompatible, and the microstructure should provide a suitable niche for cell, survival and proliferation as well as cell functionality. Furthermore, the implant of bio-materials is usually lodged in tissues where long time engraftment and lower immunogenicity are required [59].

Researchers showed that intraperitoneal transplantation of alginate-encapsulated “rat hepatocytes” could provide sufficient metabolic support to rescue an animal models with acute liver failure without immunosuppression up to 7 days [60]. More recently, human-induced pluripotent stem cell-derived hepatocyte-like cells (iPSC-HLC) were co-cultured with human stellate cells and encapsulated in alginate beads. This study showed improved differentiation efficiency of induced pluripotent stem cells (iPSCs) compared to the 2D monoculture conditions. Furthermore, the mentioned structure was implanted in immunocompetent mice for 24 days without any immune rejection [61].

In addition, acellularized ECM derived from liver could be used for cell encapsulation too. In fact, the rich content of growth factors in the ECM is important to provide proper interactions between the incorporated cells and surrounding ECM. Thus, using specific ECM can cause a remarkable effect in terms of better maintenance of encapsulated liver cells [62]. Despite recent promising results, this technology needs more validation for long-term in vitro maintenance and in vivo transplantation for clinical applications.

The major challenges in cell encapsulation technology are the risk of immunogenicity of the bio-materials and toxicity of particular components used for crosslinking. However, promotion of this field needs progress in several aspects, including more research in particular liver disease models, reduction and modification of fibrogenesis reaction during inflammation, and improvement of neovascularization through the model structure [47,63].

### 4.3. 3D Bio-Printing in Liver TE

3D bio-printing technology, as a multidisciplinary approach, benefits from chemistry, material science and biology [64]. Proper spatio-temporal status and polarity of cells as well as effective cell-cell and cell-ECM interactions could be provided using 3D bio-printers. 3D bio-printers can use different materials and structure them based on a computer-aided design (CAD) [65].

3D bio-printing technique aims to fabricate biomimetic self-assembling constructs and can use micro-tissues (or spheroids) as building blocks. However, solid organs, like the liver, are probably the most difficult ones to print because of their complex vascularization and innervation pattern [66].

During bio-printing process, live cells are suspended in a hydrogel solution, namely bio-ink. The bio-ink could be cross-linked during or immediately after the bio-printing process, to shape the final architecture of the designed construct. The hydrogel-based bio-inks may be made from natural or synthetic biomaterials, or a combination of both as hybrid materials. Natural biomaterial-based bio-inks include: alginate, gelatin, collagen, fibrin, fibronectin, gellan gum, hyaluronic acid, agarose, chitosan, silk, acellularized extracellular matrix, cellulose, etc. The synthetic bio-inks may include: polyvinylpyrrolidone (PVP), polyethylene glycol (PEG), pluronic polymers [67,68], etc. The ideal bi-oink should have the proper physiochemical properties, such as suitable mechanical, rheological, chemical and biological ones [69]. A practical biomaterial for 3D bio-printing is usually a biocompatible substance, which should be easily manipulated and it could maintain or even enhance cell viability and functions [70]. Different types of 3D bio-printing technologies have been introduced so far, including ink-jet-based bio-printing [71], laser-assisted bio-printing [72], extrusion-based bio-printing [73], stereo-lithography-based bio-printing [74] and microvalve-based bio-printing [75] and many other novel emerging technologies [76] (Table 2). Among these technologies, probably extrusion-based bio-printing has been the most widely used one to construct living 3D tissues and organs [77]. The first report using bio-printer was launched by Klebe in 1988, in which biomaterials such as collagen and fibronectin were printed while the hydrogel contained fibroblasts [78].

Later, Chang et al. used alginate as a bio-ink and designed a microchip model for drug metabolism studies. In this study, they used multi-head deposition system (MHDS) that carried out a layer-by-layer deposition of HepG2 cells and alginate simultaneously, then, integrated the 3D bio-printed construct into a microfluidic system [79]. In 2015, gelatin-alginate-fibrinogen-based hydrogel was used as a representative matrix model for natural liver ECM. The parenchymal and non-parenchymal cells were successfully embedded in this hydrogel. The results showed increased hepatocyte viability in 3D co-culture and enhanced drug metabolism [80].

Recently, a novel co-culture system using bio-printed tissue constructs seeded with primary hepatocyte, hepatic stellate and endothelial cells, has been described that successfully mimics liver fibrosis condition [81]. In 2016, a 3D microscale hexagonal architecture was printed using hydrogel in which, hiPSCs-hepatic progenitor cells (HPCs), human umbilical vein endothelial cells and adipose-derived stromal cells were embedded. This 3D model showed a practical phenotype and increased the physiologic function of cells over the weeks [74]. In 2017, a study demonstrated a method for fabricating scalable liver-like tissue by fusing hundreds of liver bud-like spheroids. Such fabricated liver-like tissue exhibited self-organization ex vivo and was successfully engrafted in rat liver. This was a new method for transplantation of ex vivo generated organoids [82]. In fact, bio-printing technology facilitates automated and high-throughput fabrication of sophisticated and controlled 3D structures. Thus, combining them with bioreactors may lead to the realization of next-generation organ-on-a-chip platforms [83].

In conclusion, 3D bio-printing is a promising technology in the field of bio-artificial organ generation, which may overcome various limitations encountered in different models [66] and improve maturation of hepatocyte like cells (HLCs) [75]. Furthermore, this technology could preserve ex vivo hepatocyte function and maintenance [71]. Also, thanks to the multi-nozzle 3D bio-printers and novel biocompatible polymers, the artificial organs could be more similar to the original tissue compartments [77].

**Table 2:** List of studies on liver 3D bio-printing for drug screening and toxicity.

Printing Technique	Bioink	Cell Type	Applications	Ref.
**Extrusion-based**	Alginate	HepG2	Drug pharmacokinetic studies	[79]
	Matrigel	HepG2 and “non-malignant mammary epithelial cell line H184b5f5 M10”	Pro-drug conversion	[84]
	Decellularized matrix-based bio-inks	PHH, primary human stellate cells, primary human Kupffer cells	Drug and toxicology screening	[85]
	Gelatin-alginate-fibrinogen hydrogel	PHH and adipose-derived stromal cells	Drug screening	[80]
	GelMA (Gelatin methacrylate)	HepG2/C3A	Toxicity assessment	[83]
	Alginate	Mouse iHep	Cell therapies and drug discovery	[86]
**Stereolithography-based**	GelMA/Glycidyl methacrylate-hyaluronic acid (GMHA)	hiPSC-HPS/HUVEC/adipose-derived MSCs	Early personalized drug screening and liver pathophysiology studies in vitro	[74]
**Inkjet-based**	Galactosylated alginate gel (GA-gel)	Mouse primary hepatocyte	preservation of functions and polarity in hepatocytes	[71]
**Microvalve-based**	Alginate	hPSC	Producing organs or tissues from patient specific cells for animal-free drug development and personalized medicine	[75]

PH: primary hepatocyte. PHH: (primary human hepatocyte.), HUVEC: (human umbilical vein endothelial cells.), hPSCs: (human pluripotent stem cells).

### 4.4. Microfluidic Systems in Liver TE

Organ-on-a-chip technologies are microfluidic systems that can recapitulate in vivo structures. These are systems, with or without perfusion, in which lobular or spheroid-based structures mimic a minimized environment in order to build functional units [87]. This promising point has attracted attention of many pharmaceutical companies. Up to now, at least 28 organ-on-a-chip companies have been registered in less than 7 years [88]. Mimicking hepatic structure and complexity is one of the reliable approaches in this field. Liver-on-a-chip systems have been shown to be able to predict possible toxicity and improve the sensitivity of certain drugs which are comparable with in vivo data [89]. One study developed an in vitro liver sinusoid chip by integrating four types of primary murine hepatic cells, including parenchymal and non-parenchymal liver cells, into two adjacent fluid channels separated by a porous permeable membrane. This microfluidic chip replicated liver physiological cell composition, microscopic architecture and mechanical microenvironment [90]. Spheroid-based microfluidic model is another approach that overcomes many problems of static cell culture systems. In 2016, a model was developed using bio-printing hepatic spheroids encapsulated in a hydrogel scaffold in a microfluidic device for drug-induced toxicity [91]. In another study, a spheroid-based model was established using co-cultivation of rat hepatocytes and hepatic stellate cells to prolong hepatic functions under chip culture condition [92].

A worthy liver-on-chip platform was reported by Lee et al. and it mimics sinusoidal and hepatic cord-like structures [93]. Some studies used hepatocytes 2D culture in liver-on-chip hepatocytes cultured in a 2D monolayer on top of a porous membrane sandwiched between two micro-channels. These systems allow hepatocytes to associate with other cells like endothelial cells [93,94].

In a recent study, researchers designed a very large-scale liver-lobule (VLSLL) on-a-chip device that provided a micro-physiological niche for hepatocytes [95].

Even though liver-on-a-chip is still in its early phases of development, recent progresses in the prediction of drug toxicity are highly promising.

## 5. Extracorporeal Liver Devices: Artificial and Bio-Artificial Devices

Engineered extracorporeal liver devices (ELD) have been designed and developed to improve or replace lost metabolic liver functions, essential in patients with decompensated liver diseases. This strategy could significantly increase the chance of receiving a compatible organ for patients stagnating in waiting lists [96].

Since accumulation of toxins is considered the main reason for liver failure, detoxification processes using biological or non-biological systems could be a promising option.

ELD categorized into two parts: Artificial liver devices (ALDs) and Bioartificial liver devices (BAL), that explained more in the next sections.

### 5.1. Artificial Liver Support Systems

Artificial liver devices (ALDs) usually work based on simple principles such as albumin dialysis, membrane filtration and the use of adsorbent columns to remove toxins [97]. For the first time, in 1988, charcoal hemoperfusion helped patients with fulminant hepatic failure [98]. Advanced supporting systems such as Molecular Adsorbents Recirculating System, (MARS, Gambro, Sweden) developed by Stange et al. [99] and Fractionated Plasma Separation, Adsorption and Dialysis system (FPAD, Prometheus, Fresenius Medical Care, Bad Hamburg Germany), have been clinically used to eliminate protein-bounded bilirubin and bile acids. Single-pass albumin dialysis (SPAD) and selective plasma filtration therapy (SEPET^TM^ Arbios systems, Allendale, New Jersey, USA) are other examples for advanced liver supporting systems [100]. MARS and SPAD use dialysis-based techniques in which, blood flow stream passes through a highly selective/small porosity (<50 kDa), high-flux membrane against an albumin-containing solution [101]. In contrast, in plasma adsorption techniques, such as Prometheus system, non-selective membranes (approximately 250 kDa) are used and there is no parallel dialysate circuit [102]. Treating patients with Prometheus supporting system adjusted serum levels of conjugated bilirubin, ammonia, creatinine, bile acids, and urea and improved blood pH [97] (Table 3).

### 5.2. Bio-Artificial Liver (BAL) Support Systems

BAL devices are hybrid systems composed of functional hepatocytes alongside with artificial membranes, in order to provide active detoxification and biosynthetic hepatic functions [101]. More than 30 cell-based support systems have been launched since 1987, and more than 14 BAL systems have been used in clinical trials [103] (Table 4).

BAL devices have some advantages over ALD, since they offer active exchange of biomolecules and detoxification. Widespread application of BALs faced many challenges that eventually limited their broad application. The main challenges are: i) a reliable cell source, ii) complicated and expensive technology, and iii) risk of xeno-contamination while using porcine cell lines [104]. BAL devices require a minimal number of 10^10^ functional hepatocytes. This number represents almost 10% of total liver mass in adults [105]. Different cell sources that have been used in BAL devices include: immortalized human hepatocyte cell lines [106], primary porcine [107] and human hepatocytes [108]. In vitro-generated HLCs from different pluripotent cells (embryonic stem cells (ESCs), iPSCs), could be other sources. Because of the low functionality of HLCs compared to primary hepatocytes, porcine hepatocytes are the most common cells used in BALs. Although the maintenance of porcine hepatocytes is acceptable in culture condition, the risk of xeno-infection and polymorphic metabolic incompatibility made them inappropriate cells in BALs [109,110].

**Figure 2:**
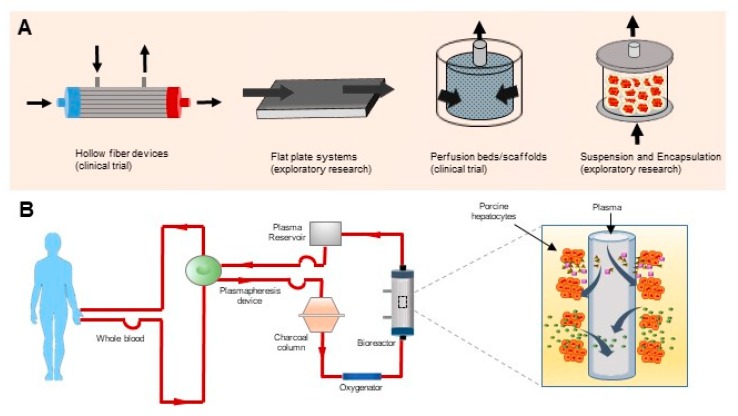
(**A**) Different platforms for “Extracorporeal Liver Assist Device”. (**B**) HepatAssist system. A hollow fiber bioreactor containing various parts e.g., bioreactor, charcoal filter, membrane oxygenator, and pump.

Based on device configuration, different types of BALs are available. Hollow fiber devices, packed beds, flat plate systems and encapsulation-based reactors are common examples of BALs (Figure 2A).

Hollow fiber systems are the most common BAL in which, hepatocytes are located within a cartridge. The hepatocytes adhere to hollow fiber membranes which play as an scaffold for cell attachment and compartmentalization [111]. Up to now, several hollow fiber-based devices have been developed. Extracorporeal Liver Assist Device (ELAD; Vital Therapies Inc., San Diego, CA, USA) and HepatAssist^®^ (Alliqua Inc., Langhorne, PA, USA) are two important examples [104].

In ELAD, a hollow fiber membrane separates the functional cells from the patient’s plasma and integrated charcoal absorber and the oxygenator. Human hepatoblastoma cells have been used to support detoxification and maintain the oxygen supply to the functional cells [112]. Clinical trials accomplished using the ELAD are limited to a few pilot studies where safety was the only primary outcome. The efficacy resulted in a limited number of studies results, and no reliable results were reported in randomized trials [96].

HepatAssist^®^, as the first device that was applied in a phase II/III clinical trial. It consists of an extra-capillary compartment of a hollow fiber bioreactor which contains cryopreserved porcine hepatocytes (Figure 2B), where patient’s plasma is separated and circulated through a charcoal filter and oxygenator. Several studies reported safety of this device, however positive impact on the survival rate was not demonstrated [96].

However, designing hollow fiber-based devices faced several challenges, such as, successful oxygen/nutrients delivery, shear-induced cell damage and clinically-relevant scale-up protocols [113].

Moreover, it has been shown that current ALDs and BALs have potential limitations, and preliminary data supporting the use of HepatAssist was not promising. In this regard, further research is recommended to find other functional cell sources, like genetically-modified liver cell lines, humanized pig hepatocytes, and hepatocyte spheroids [104,114].

## 6. In Vivo and In Vitro Modeling for TE of Liver Diseases

Technology demonstrated a great promise to mimic in vivo conditions and provide a specific microenvironment for ex-vivo culture of isolated primary cells. Developing a functional liver-on-a-chip or micro-platform-based bioreactor could provide controlled and patient-specific microenvironment. Toxicology studies and drug screening will also benefit from such sophisticated culture conditions [17]. In vitro culture condition can help us to understand the regulations in the establishment of hepatic metabolic zonation. Substantial advances are developing in metabolic liver zonation to study hepatocyte functions and zone-specific toxicity [115]. The zonation studies can be performed in microtiter plate in static cultures [116], bioreactors [117] and microfluidic systems [118].

In the past decades, animal models have been routinely used in biological experiments. The use of physiologically relevant models is of crucial importance in preclinical development. Among various experimental animals, rodents have been the best choice for modeling human liver diseases. Liu and his colleagues divided animal models into two groups to study liver diseases: i) those generally used for studying mechanisms of liver fibrosis, and ii) those used to mimic specific chronic liver diseases (CLDs) including autoimmune and cholestatic liver diseases, chronic viral infection, nonalcoholic fatty liver diseases (NAFLD) and alcoholic liver diseases [119] (Table 5).

Using animal models in research faces many challenges, such as time and high costs as well as ethical concerns [147]. Moreover, the most important challenge in the use of animal models is that they often fail to predict the clinical efficacy of therapeutics due to different pharmacokinetics, pharmacodynamics and inter-species genetic and metabolic variations [148,149].

Over the years, various liver-derived in vitro models have been developed to investigate the effects of drugs and chemicals [150]. Some of these models include 2D cultures [151], spheroid culture [152], sandwich cultures [43], hollow-fiber bioreactors [153], micro-patterned co-cultures [154], microfluidic liver biochips [155] and bio-printers [156] as already described in details.

However, traditional 2D cultures cannot maintain drug metabolism gene expression for more than 24-72 h and have a low sensitivity to drugs [157]. In 2015, researchers designed a micro-pattern of iPSC-HLC that was in co-cultured with murine embryonic fibroblasts. This system was used as a model to drug toxicity assays [158] and later upgraded to use primary human hepatocytes in co-culture with fibroblasts, where in vitro hepatic life cycles for hepatitis B and C viruses and the malaria parasites *Plasmodium falciparum* and *Plasmodium vivax* were recapitulated [154].

In the recent decade, 3D models became popular because of their abilities to mimic in vivo environment. This feature is essential for drug testing since micro-environmental properties could affect behaviors and functions of primary cells [159,160]. Landry et al. developed some of the first spheroid structures [161].

Hepatocyte-ECM interaction provides polarity in hepatocytes and can be modeled as a sandwich culture by culturing hepatocytes between the two layers of ECM. Such platform has served as a tool for analysis of long-term hepatocytes function and drug-induced toxicity assays [162,163,164].

In recent years, a considerable effort has been made to improve 3D human-based microsystems to organize cells in a controllable manner [148]. In 2016, one scalable 3D PHH spheroid system was developed to model drug-induced liver injury (DILI) [165].

Besides organoid and spheroid-based culture, there is one main category of dynamic in vitro models, organ-on-a-chip. These platforms utilize advanced micro-fabrication techniques to create miniature structures that mimic structure and functions of the organ in vitro [87,166]. Table 6 lists common in vitro models used in drug toxicity.

## 7. Conclusion and Future Remarks

By now, OLT has been known as the only effective treatment in end-stage liver diseases, limited by the shortage of donated organs. Therefore, replacement of this treatment with accessible, reliable and applicable methods is urgently needed. Liver TE and regenerative medicine are two modern promising multi-disciplinary fields to improve liver failure therapies. Technical approaches in liver TE are based on different methods including organ acellularization, in vitro modeling, artificial liver, cell encapsulation, 3D printing and organ on a chip. A recent breakthrough in technology is 3D bioprinting that has enabled to print functional artificial liver micro tissues for transplantation instead of real organ transplantation. A suitable ECM or synthetic components which have appropriate topography and biomechanical properties can facilitate hepatocytes colonization, migration, differentiation, proliferation and cell polarity. Primary human and porcine hepatocytes, immortalized cell lines and stem cells and human cell lines have been proposed in liver TE field. Taken together, different findings proposed that a suitable cell source that is cultured in 3D platform with acceptable scaffold and using reliable technology such as 3D printing, could generate a functional liver model for transplantation and aim to other purposes such as drug screening, diseases modeling, precision medicine and so on.

## Figures and Tables

**Table 1:** Common types materials used in 3D cultures, and their advantages and disadvantages.

Type of 3D Culture	Cultivation Technique/Coating Material	Production Technique	Advantages	Disadvantages	Ref.
**Hydrogel based Scaffold**	Collagen Sandwich, Collagen Gel/Isolated from rat tails	Gel formation by crosslinking of the water-soaked collagen–fibers	a) Containing collagen type I b) Maintenance of hepatocytes polarity including transporter activity	a) Reduced exchange of nutrients and waste products between cells and medium b) Dead cells were not removed within the matrix C) Disruption of living cells by proteases released from dead cells	[41]
	Matrigel/ECM proteins extracted from mice Englebreth-Holm-Swarm tumors	Cold Matrigel is mixed with medium and plated between 2 and 6 °C as fluid solution. Temperatures ≥ 10 °C results in a solid gel formation	a) Cell polarity preserved b) Containing various ECM proteins and growth factors c) Promotion of cell differentiation	a) The same disadvantages as described for collagen b) The components of the Matrigel are not well defined	[42,43]
**Scaffold**	Decellularized Human Liver as a Natural Scaffold	Tissue was decellularized, remaining ECM was used as scaffold for culture	a) Perfectly represents the structural features as well as the biochemical components of the human liver matrix	a) Elaborate production b) Limited availability of donor tissue	[44]
	Cryogel/PHEMA, Bis-Acrylamide, Alginate, Gelatin, Collagen	Monomers are frozen in aqueous solution with crosslinking agents. Ice crystals form, which remains after polymerization and thawing as pores in the scaffold matrix	a) Simple preparation b) Create various pore sizes and stiffness	a) Difficult standardization of the manufacturing process b) Variation in scaffold parameters possible only in certain range	[27,29]
	Electrospinning/Natural or synthetic polymer solutions	electrostatic fiber formation which utilizes electrical forces to produce polymer fibers	a) Relatively high standardizable b) Using different materials c) Using different fiber strengths and degrees of intertwining adjustable	a) Generating solid tissue structure during electrospinning intertwined fibers	[28,45]
	3D printing/Natural products like gelatin and 1-ethyl-3-(3-dimethylaminopropyl) carbodiimide (EDC) and N-hydroxy succinimide (NHS) for crosslinking	Scaffold was printed by using a 3D printer	a) Uniform and reproducible b) Reduction of user error c) Precisely adjustable scaffold pore size d) interconnectivity and controlled geometry	a) Requires elaborated equipment b) High standardization results in lacking of representation of the biological variability c) Generating pores with many different sizes is difficult	[46]

ECM: extracellular matrix. PHEMA: Poly 2-hydroxyethyl methacrylate.

**Table 3:** Artificial liver support devices.

**(A) Non–Albumin-Based Devices**	
**Method**	**Brief Explanation**
Hemodialysis	In 1958 Kiley et al. described the symptomatic and clinical improvement in form of improved neurological status in four of the five patients of ammonia intoxication treated by hemodialysis. However, no benefit was noted in long-term survival of these patients.
Charcoal hemoperfusion	Initially used in the treatment of barbiturate poisoning, charcoal hemoperfusion has been shown to remove many water-soluble molecules associated with encephalopathy in hepatic failure patients.
Hemodi-absorption	This is a procedure that has the capability of removing toxins of less than 5 kDa. These include aromatic amino acids, glutamine, mercaptans, benzodiazepine-like substances, false neural transmitters, ammonia, and manganese.
Plasma exchange TPE (Therapeutic Plasma Exchange) HVP (High Volume Plasma exchange)	Plasma element is separated from cellular blood components of blood by using a hollow fiber filter made of cellulose diacetate and polyethylene membrane or other synthetic materials.
Hemodiafiltration	This is a combination of hemodialysis and hemofiltration. Hemodialysis is useful for removing molecules which are less than 5 kDa and hemofiltration can remove molecules in the 5–10 kDa range. A high-performance membrane such as a large-pore sized poly methyl methacrylate (PMMA) membrane is performed.
**(B) Albumin-based systems**	
**Company**	**Brief explanation**
MARS^®^ (molecular adsorbent recirculating system)	Uses a high-flux hollow-fiber hemodiafilter and albumin as the acceptor molecule for albumin-bound toxins within the extracorporeal circuit
Prometheus	Based on an albumin-permeable polysulfone membrane, which enables the patient’s albumin fraction to pass into a secondary circuit in which the direct purification from albumin-bound toxins by different absorbers (that is, anion exchanger and neutral resin) takes place.
SPAD (single-pass albumin dialysis)	It uses a standard continuous renal replacement therapy system without any additional columns or circuits. Blood is dialyzed against a standard dialysis solution with the addition of 4.4% albumin in the dialysate.
SEPET (selective plasma filtration therapy)	Combines aspects of fractionated plasma separation, adsorption and single-pass albumin dialysis. The fractionated plasma passes through an albumin-permeable size-selective membrane.
BioLogic-DT (later Liver Dialysis System™ [HemoCleanse, Lafayette, IN, USA])	Based on a cellulosic plate dialyzer with a suspension of powdered charcoal and cation exchangers as dialysates, is no longer marketed.

**Table 4:** Commercially available bio-artificial liver devices (BAL).

Bio-artificial Liver Systems		
Company	Bioactive Functional Cells	Explanation
HepatAssist	Cryopreserved Porcine hepatocytes (7 × 10^9^ cells)	Plasma is separated from blood cells and then the plasma is circulated through the bioreactor after first passing through a charcoal filter and an oxygenator.
ELAD^®^ (Extracorporeal Liver Assist Device)	Hepatoblastoma cell line HepG2-C3A (200–400 g)	The cells are isolated from the patient’s plasma by hollow-fiber membranes. An integrated charcoal absorber, and a membrane oxygenator supports detoxification and maintains the oxygen supply of the cells.
AMC-BAL (Amsterdam Medical Center-Bioartifcial Liver device)	Porcine hepatocytes (10–14 × 10^9^ cells)	The plasma is in direct contact with the cells, lead to better mass exchange between cells and the patient’s plasma.
MELS (Modular Extracorporeal Liver Support)	Human hepatocytes (up to 650 g)	The bioreactor is composed of a three-dimensional matrix interwoven with bundles of hollow fibers. The hollow fibers have a molecular cutoff weight of 400 kDa and used to perfuse patient’s plasma adjacent to the functional hepatocytes.
BLSS (Bioartificial Liver Support System)	Porcine hepatocytes (70–120 g)	Whole blood, rather than plasma, is passed through the fibers after warming and oxygenation.

**Table 5:** Conventional in vivo models used for liver diseases.

Main Models	Models in Specific Diseases	Methods/Agent	Ref.
**Classical Animal Models**	Liver Fibrosis	CCl_4_	[120]
		TAA	[121]
		DEN and DMN	[122]
	Experimental obstructive cholestasis	Common bile duct ligation	[123]
	Genetically engineered mice	TGF-β1 transgenic mice	[124]
		PDGF transgenic mice	[125]
		Bcl-xL^−/−^ mice	[126]
**Animal Models of specific Liver Diseases**	Primary Sclerosing Cholangitis	DDC diet	[127]
		Abcd4^−/−^ mice	[128]
		Cftr^−/−^ mice	[129]
	Primary Biliary Cholangitis	Spontaneous Mouse Models	[130]
		Chemical Xenobiotics–Immunized Mice	[131]
	Autoimmune Hepatitis	Concanavalin A Hepatitis	[132]
		BALB/c Strain TGF-β1^−/−^ mice	[133]
		NTx-PD-1^−/−^ Mice	[134]
		Alb-HA/CL4-TCR Mice	[135]
		Ad-2D6–Infected Mice	[136]
	Alcoholic Liver Diseases	Acute binge ethanol–feeding model	[137]
		Liquid diet model	[138]
		Intragastric ethanol infusion model	[139]
		Chronic plus binge ethanol feeding model	[140]
	Nonalcoholic Fatty Liver Disease	Genetic models	[119]
		Dietary models	[141]
	Hepatitis C	Inducible-HCV transgenic mice	[142]
		Genetically humanized mouse models	[143]
	Hepatitis B	Animals That Permit HBV Infection and HBV-Associated Viruses That Infect Animals	[144]
		HBV Transgenic Mice	[145]
		Human Hepatocyte Chimeric Mice	[146]

CCl_4_, carbon tetrachloride; TAA, thioacetamide; DEN, diethylnitrosamine; DMN, dimethylnitrosamine; TGF-β1, transforming growth factor beta; PDGF, platelet-derived growth factor; Cftr, cystic fibrosis transmembrane conductance regulator; HCV, hepatitis C virus; HBV, hepatitis B virus; DDC, 3,5-diethoxycarboncyl-1,4-dihydrocollidine.

**Table 6:** Common hepatic in vitro models for drug toxicity studies.

Models	Cell Type/Culture Condition	Applications	Advantages	Disadvantages	Ref.
**Hepatocyte sandwich culture**	Hepatocytes (PHH)	A model to study hepatobiliary transportation and cholestasis (Drug-induced) liver injury	a) Maintenance of cell polarity and polygonal morphology b) Formation of functional bile canaliculi	a) Decreasing metabolic enzyme activity b) losing liver functionality, morphology and phenotype in long-term cultures	[162,163,167,168]
**3D models**	HepG2	Drug toxicity	a) Providing cell-cell interaction b) Maintenance of cell polarity c) Formation of functional bile canaliculi-like structures	a) Lack of many phenotypic and functional characteristics of the liver tissue	[169,170]
	HepaRG	Hepatotoxins screening A model to study drug-induced fibrosis	a) Formation of bile canaliculi-like structures b) Expression of functional bile acid transporters metabolic enzymes	a) Lack of many phenotypic and functional characteristics of the liver tissue	[171,172,173]
	Hepatocytes (PHH)	Drug toxicity assessments A model to chronic drug assessment	a) Increased CYPs activity b) Long term functionality	a) No bile canaliculi	[165,174,175]
	Stem cell-derived hepatocytes	Drug toxicity testing	a) Creating an accessible and useful model systems for viral and inherited metabolic disorders	a) Low expression of liver specific genes in metabolism b) Limited results regarding toxicology	[176]
**Organ–on a chip platforms**	Co–cultured Micro patterned cells	Drug toxicity tests	a) Preserved zonation b) Continuous perfusion of medium	Batch-to-batch variation of ECM substrates	[176,177,178]
	Perfused multiwall plate	Drug metabolism and drug toxicity assays	a) Facilitated nutrient exchange b) Efficient shear stress	a) Need more functional cells b) Consuming more culture media	[179,180]
	Microfluidic liver biochips	Toxicity assays	a) Facilitated nutrient exchange b) Efficient shear stress c) Mimicking in vivo environment, i.e., hexagonal structure	a) Complex system to establish and maintenance b) Sampling is difficult	[181,182]
**3D bioprinting**	3D liver bioprinting	Toxicity assays	a) Using bioink b) Sophisticated shaping	a) Complex system to establish and maintenance	[74,156]

CYPs, cytochromes P450.
